# Study on the Rehabilitation Therapist Estimation Under Institutional Perspective by Applying the Workload Indicators of Staffing Needs in the Aging Context

**DOI:** 10.3389/fpubh.2022.929675

**Published:** 2022-06-16

**Authors:** Qi Jing, Yang Xing, Mingxue Duan, Peiwu Guo, Weiqin Cai, Qianqian Gao, Runguo Gao, Lihong Ji, Jun Lu

**Affiliations:** ^1^School of Management, Weifang Medical University, Weifang, China; ^2^China Rehabilitation and Health Institute, Weifang Medical University, Weifang, China; ^3^Weifang People's Hospital, Weifang, China; ^4^School of Public Health, Weifang Medical University, Weifang, China; ^5^School of Public Health/China Research Center on Disability Issues, Fudan University, Shanghai, China

**Keywords:** WISN, rehabilitation, therapist, aging, China, public health

## Abstract

**Background:**

The need for rehabilitation therapy has increased dramatically with the aging of the population, the prevalence of non-communicable diseases, and the increase in the number of disabilities. Rehabilitation therapists are crucial to provide high quality rehabilitation therapy; however, there is a significant shortage of these professionals in China. One of the effective strategies to address this challenge is using the norm of the workforce for rehabilitation therapy, which is an index for assessing the personnel required in a facility. This research aimed to create a rehabilitation therapist-required norm under institutional perspective in Shandong Province, China, based on the Workload Indicators of Staffing Needs (WISN) method, which was created by the World Health Organization (WHO) in 1998 to analyse staff utilization at various levels of the health care system.

**Methods:**

We conducted descriptive and quantitative research from October to November 2020 in the rehabilitation department of a tertiary hospital in Weifang City, China. Focus groups, online interviews, and document reviews were conducted to gather data and calculations of the WISN method performed.

**Results:**

Admission assessment, pre-treatment evaluation, rehabilitation therapy, post-treatment evaluation, and health education for patients were identified as the main priority group activities. Interviews and analysis of documents summarized five factors related to rehabilitation therapists' health service activities. In this study, the annual working time of each therapist was 1,776 h per year. The WISN method calculations showed that the norm of rehabilitation therapists in this tertiary hospital was 23 therapists. As the department had 13 therapists, there was a shortage of 10 therapists based on the WISN calculation, with a ratio of 0.57, which represented the actual compared to the ideal number of therapists.

**Conclusion:**

Workload pressure was high for therapists in this tertiary hospital. This model revealed a demand for ten more therapists in the rehabilitation department. The WISN method can help hospital administrators in therapist workforce monitoring, including in regard to therapists. Therefore, the WISN method should be embraced as part of hospital human resource planning and recruitment strategies to meet increasing rehabilitation needs.

## Introduction

According to the World Health Organization, approximately 15% of the world's total population has had some type of disability (1). With an increase in the aging population and the prevalence of non-communicable diseases, the number of disabilities will substantially increase (2). Rehabilitation is the action of restoring a person to health or normal life through training and therapy after imprisonment, addiction, or illness and is an essential health service for people with a wide range of health conditions during all phases of acute, sub-acute, and long-term care.

One of the health targets of Sustainable Development Goal 3 (SDG-3) and a global strategy for the health workforce is to increase training and retention of health personnel, a central element of health services (3). There is global consensus on the need to strengthen the rehabilitation sector in health systems, including the development of new rehabilitation human resource planning (4). As an important category of the health workforce, rehabilitation therapists provide comprehensive rehabilitation assessments and apply for entire rehabilitation programmes in line with all phases of patients' acute, sub-acute, and long-term conditions (5).

China is a developing country with the second largest economy worldwide and is facing multiple challenges, including an aging population, high incidences of chronic diseases, and an increased number of people with disabilities (PDs); therefore, the need for rehabilitation services is rapidly increasing (6). At the end of 2019, the population aged 60 years and above numbered nearly 254 million in China, accounting for 18.1% (7) of the world's older adult population. According to the sixth National Census and the second national sample survey of PDs, the actual population of PDs was almost 85 million, of which three-fifths had rehabilitation needs (8).

The Workload Indicators of Staffing Need (WISN) approach could provide a device to discuss the staffing norms or plans for rehabilitation therapists (9). The WISN is a workforce-planning device that considers data when calculating staffing requirements for health service supply. The methodology uses the data available in health information systems and provides options for closing gaps in workforce availability. The data are analyzed using specific formulas (more details on this are provided in the Methods section). The WISN method may be able to determine the specific types of health workforce required to provide services in a health institution as well as assign new personnel or redeploy existing personnel dependent on workload pressure and redistribution. In recent decades, the WISN method has been proposed in some studies for staffing the health workforce, including nurses (10), infection preventionists, general practitioners, and specialists (11). The WISN is also a dynamic tool that can be repeated on a regular basis to enhance the adequacy and distribution of human resources for health facilities. The tool has been used in many countries, including Ghana (12), Kenya (13), India (14), and Bangladesh (15).

In 1998, the World Health Organization (WHO) proposed a methodology for adjusting staffing levels to achieve equitable and optimal distribution of health facility staff from local to national levels (16). The WHO intends to broaden the evidence from the application experiences of WISN studies in diversifying contexts to assist human resource planning for health. The aim of the study is to explore WISN for rehabilitation workforce management at the facility level, owing to the lack of measurement tools for staffing requirements of rehabilitation therapists. There is no consensus on rehabilitation therapist staffing norms in China, and there is a lack of academic concern regarding this issue. This study seeks to explore some of the challenges encountered in assuring the quality of services, the promotion of constant service delivery, and the productive management of personnel at the hospital.

## Materials and Methods

### Scope and Setting

This research was conducted from October to November 2020 in the RD of a tertiary educational hospital in Shandong Province, China. The RD is the key municipal department for rehabilitation in Weifang City. There are 12 beds and 10 physicians (one chief physician, two associate chief physicians, six attending doctors, and one physician assistant), 22 staff nurses, and 13 rehabilitation therapists (including 11 who are focused on physical rehabilitation, one on occupational rehabilitation, and one on speech rehabilitation).

The RD activities comprised three main categories: neurological rehabilitation, rehabilitation of osteoarthritis, and other rehabilitation. Neurological rehabilitation, which accounted for 60–70% of the total RD visits, included rehabilitation for patients with conditions such as a stroke, traumatic brain and spinal cord injury, peripheral nerve injury, Parkinson's disease, and cerebral palsy. Visits by older patients with a stroke or traumatic brain and spinal cord injury accounted for 50% of visits in this category. Rehabilitation of osteoarthritis, which accounted for 30–40% of the total RD visits, included various types of osteoarthritis, cervical spondylosis, periarthritis of the shoulder, lumbocrural pain, scoliosis, post-fracture dysfunction, dysfunction after tendon and joint injury, sports trauma, rheumatism and rheumatoid arthritis, post-joint replacement, and so on. Other rehabilitation included rehabilitation for chronic pain, physiotherapy for acute and chronic inflammation (facial features, skin, breathing), and sub-health consultations, rehabilitation, and non-surgical treatments.

The annual number of patients who received rehabilitation therapy for the year 2019 was 21,840. If the same therapists perform the same activity multiple times throughout the day, the number of times that specific activity is performed is used to calculate the workload. We used the list of template tables provided in the WISN user's manual illustrated by the WHO (19) to guide each step of the research.

### Data Collection

Qualitative and quantitative data were collected. Qualitative data were obtained from interviews with the human resources officer and the head of the rehabilitation department (RD). Quantitative data were obtained by surveying therapists in the RD using a questionnaire with WISN indicators; namely, activity standard, allowance factor, available working time (AWT), standard workload, and workload component were chosen to determine the staffing requirements of rehabilitation therapists for the selected tertiary hospital in Shandong Province, China. The data needed include the main activities by selected groups during daily duties at the various levels of health service. Two types of results—differences and ratios—are provided by the WISN method. The former is the size of the staff shortage or surplus for the particular staff category, and the latter represents the measure of the workload pressure of the individual staff member.

A hospital administrator and experienced therapists who had worked for at least 5 years administered structured individual and group interviews with therapists to identify the health service activities, support, and additional activities performed by each therapist. The time taken by a therapist in the RD to perform each drafted rehabilitation therapy service activity was calculated by the head of rehabilitation therapy and a hospital administrator from the department of human resources. The time taken for each activity was observed by the research team and from self-assessments of experienced therapists in the RD. Because direct observation of support and additional activities was not possible, the time spent performing each related activity was calculated based on an approximation given by the therapist through online interviews. Administrative records were used to access annual service statistics by our team members.

### Data Analysis

Analyses of the workload and the staffing requirements were performed based on the WISN approach (Table 1).

**Table 1:** WISN steps.

**Analysis stage**	**Contents**
STEP 1	Determining the priority group. China is an aging society; the need for rehabilitation has increased dramatically; rehabilitation started late and developed slowly, and a complete rehabilitation system was not established.
STEP 2	Estimating AWT. AWT = [A–(B + C + D + E)] × F. Time per year (365 days) minus [public holidays (13 days) + weekends (104 days) + average sick days (10 days) + others, such as training (16 days)]* working hours per day (8 hours)=222 days/1,776 h.
STEP 3	Defining workload components. Specialist consultation and clinical observation were conducted based on the WISN user manual.
STEP 4	Setting activity standards. Consult ICF guidelines and use expert consultation and clinical observation.
STEP 5	Establishing standard workloads. Standard workload = AWT in a year divided by unit time. Using clinical observation of the time required to serve one client per person* AWT.
STEP 6	Calculating allowance factors. CAF = 1 ÷ [1–(total CAS percentage /100)] = 1 ÷ [1–(8.25/100)] = 1.10 The final category allowance factor is 1.10.
STEP 7	Determining staff requirements based on WISN. Total required number of staff based on WISN: (A x B + C) = 23.
STEP 8	Analyzing and interpreting WISN results. The actual number of RTs is 13. Based on WISN's result of 23, the gap can be concluded as 10. Using the ratio of actual quantity to actual quantity, which is 0.57, it can be concluded that there is currently some work pressure.

*AWT, available working time; WISN, workload indicators of staffing needs*.

## Results

### The Available Working Time

Public holidays represent the days when almost everyone in China is given time off from work. We accounted for 52 weekends; sick leave, which is recorded by the head of the rehabilitation therapist's team, represents the therapist's absence due to sickness, and other leave includes training, vacation, and personal leave. Using the formula, the predicted AWT for rehabilitation therapists was 222 days or 1,776 hours in 1 year (Table 2).

**Table 2:** Possible annual working days, non-working days, and AWT.

**Items**	**Staff category**
	**Rehabilitation therapist**
Possible working days in one year (A)	365
Public holidays (B)	13
Weekends (C)	104
Sick leave (D)	10
Other leave (training, personal leave, etc.) (E)	16
Number of working hours in one day (F)	8
AWT	222 days/1,776 h

### Setting Activity Standards

The activity standard is a unit of time for a rehabilitation therapy activity of a therapist, including period and average of time performing admission assessment, pre-treatment evaluation, rehabilitation therapy, and post-treatment evaluation. The service standards and allowance standards were defined as follows: the standards of services and activities reported in the annual service statistics were termed service standards, and the allowance standards applied to activities that were not reported. The unit of time for each rehabilitation therapy activity was counted using the information collected from observations and self-assessments by rehabilitation therapists to define the activity standards (Table 3).

**Table 3:** Workload components and related time of rehabilitation therapists.

**Workload group**	**Workload components**	**Unit of time of working**
Rehabilitation therapy service activities of all rehabilitation therapists	Admission assessment	20 min
	Pre-treatment evaluation	9 min
	Rehabilitation therapy	35 min
	Post-treatment evaluation Health education for patients	14 min 20 min
		Time spent
Support activities conducted by all staff therapists	Literature review	126 min per month
	Professional learning	134 min per month
	Case discussions	96 min per month
	Other departments' ward rounds	88 min per month
	Meetings	90 min per month
	Notes and reports	138 min per month
	Follow-ups	61 min per month
		IAS (actual working time per person)
Additional activities of certain staff therapists	Supervision and teaching of therapist interns	0.92 h per day
	Continuing education	0.33 h per day
	General administration	0.25 h per day

### Calculating Allowance Factors

There are two sorts of allowance standards: category allowance standards (CAS) and individual allowance standards (IAS) (Table 4). The former is determined for support activities that rehabilitation therapists perform, while the latter is for services that are conducted only by a certain rehabilitation therapist in the RD. CAS was counted as the percentage of working time spent on a service as observed by the personnel. The category allowance factor (CAF) was calculated using the following formula:


$$\begin{array}{l} CAF=1÷[1-(\text{total CAS percentage}/100)]\\ \;=1÷[1-(8.25/100)]=1.10 \end{array}$$

The time required to perform each additional activity was calculated and multiplied by the number of rehabilitation therapists (RTs) required to perform that therapy activity per year. The time required for all rehabilitation-related activities was then added to calculate the total IAS per year. Finally, the individual allowance factor was calculated by dividing the total IAS by the total AWT of each therapist (Table 5).

**Table 4:** Calculation of category allowance standard (CAS).

	**Activity**	**Time spent**	**CAS (% of working time)**
Support activities done by all therapists	1 Literature review	126 min per month	1.42
	2 Professional learning	134 min per month	1.51
	3 Case discussions	96 min per month	1.08
	4 Other departments' ward rounds	88 min per month	0.99
	5 Meetings	90 min per month	1.01
	6 Notes and reports	138 min per month	1.55
	7 Follow-ups	61 min per month	0.69
	Total		8.25

**Table 5:** Calculation of individual allowance factor (IAF).

**Workload group**	**Workload components**	**Number of staff members performing the work**	**IAS (actual working time per person)**	**Annual IAS (for all staff conducting the activity)**
Additional activities of staff therapists	Supervision and teaching of therapist interns	4	0.92 h per day	814 h
	Continuing education	3	0.33 h per day	222 h
	General administration	2	0.25 h per day	111 h
	Total IAS in a year	1,147 h
	Individual Allowance Factor (IAF)	0.65

### Determining Staff Requirements

The total number of required RTs was calculated by multiplying the total number of personnel obtained above by the category allowance factor. Later, the individual allowance factor was added to the result. The total number of therapists required was calculated using the total required staff for rehabilitation therapist activities × CAF + year long total IAS/AWT. This is 20.12 × 1.10 + 0.65 = 22.78 = 23. Hence, 23 RTs are required for the RD in this tertiary educational hospital.

### WISN Difference and Ratio

The number of RTs at the hospital at the time of this study was 13. According to the WISN approach, the total number of RTs needed was 23; therefore, the gap was 10. In addition, the WISN ratio was 0.57, indicating that the RD rehabilitation therapist requirements were 57% fulfilled, leaving the RD 43% understaffed, which demonstrates pressure on the RTs in this situation. Based on the formula [1–WISN ratio] ^*^ 100 and the classification ranges “low” (1–29%), “high” (30–40%), “very high” (41–60%), and “extremely high” (>60%) (17), the workload pressure of the RD was very high (0.43).

## Discussion

In the aging context, the WISN tool was demonstrated to be useful in setting facility-level norms for health workforce. This WISN-based estimation did not consider other absences, such as maternity leave and therapists' participation in further learning in other hospitals. The WISN tool was previously used to help many countries develop evidenced-based staffing norms for their nurses, doctors, pharmacists, laboratory staff, and medical officers at the national, regional, or organizational level. When the WISN ratio is one, there is a balance between department needs and existing staffing; if it is higher than one, the department is stress-free, while if it is lower than one, the department is understaffed. Thus, the WISN ratio in this study showed that the therapists in the department were under severe pressure.

Globally, the education and training of RTs began earlier than in China. Physical therapists, occupational therapists, and speech therapists all have their own educational systems and job classifications. However, in China, there is only one major—rehabilitation therapy. After graduation, the students are assigned to different paths when they enter facilities such as this studied hospital. Thus, in this research, RTs included all categories of rehabilitation.

In the Evaluation Criteria for Rehabilitation Medical Service Model Hospitals (Tertiary General Hospitals; Trial Version) (18), produced by the Ministry of Health in 2012, the ratio of the total number of RTs to the total number of beds in the RD's ward should not be <1:2; those with qualifications for senior professional and technical positions should account for more than 5% of the total number of RTs, and those with qualifications for intermediate professional and technical positions should account for more than 25% of the total number of RTs. The hospital in this study has a ratio of nearly 1:1 (12 beds vs. 13 therapists). However, there were no therapists with qualifications for senior professional or technical positions and only one with qualifications for an intermediate professional and technical position. The actual number of therapists with senior professional or technical qualifications does not even reach the model criteria that were established 8 years ago. In this survey, we determined the estimation benchmark for the total needed RTs in the study hospital. Findings from this WISN survey showed that the RD in the study hospital in Weifang suffered from some workload pressure. This study provides a reference for the personnel allocation of the RD.

Our study has several limitations. The WISN approach adopts year long service statistics to determine workload; thus, the consequences depend on the veracity of the statistics. Based on an undeveloped information system, data, such as the number of patients, come from the head of RTs, where a bias could exist. We interviewed participants *via* an online questionnaire, and some questions were self-evaluated. Thus, data collection systems should be improved in the future. In addition, the observation times were limited because of the COVID-19 pandemic; it was difficult to get long-term access to the hospital. Further, the WISN approach uses the statistics of health services from the prior year; accordingly, this approach counts the total needed therapists for the previous year (19) and may not represent current needs. Further, only one hospital affiliated with Weifang Medical University was analyzed in this study, which makes it difficult to generalize the results to other hospitals; however, as this is a new concept, we hope that it can be used as a foundation for further studies.

## Conclusion

The workload pressure of therapists in the targeted tertiary hospital's RD is high in the context of aging. This department needs to be staffed to ensure contentment for therapists. The WISN approach is an uncomplicated, easy-to-use tool that can eventually estimate therapy activities and transform workload into rehabilitation therapy time substitutes for the RD. In the future, the approach may be applied to broaden the rehabilitation therapy function and blueprint staffing to strengthen an RD's performance. In short, planning for rehabilitation therapy staff can be performed utilizing the WISN approach for convenient allocation and distribution. Human resource management is a major challenge (20); with the vision of Healthy China 2030, China needs to strive to optimize its existing human resources for health, including RTs.

## Data Availability Statement

The original contributions presented in the study are included in the article/supplementary material, further inquiries can be directed to the corresponding author.

## Author Contributions

QJ and YX conducted the study design, analysis, and basic writing. YX and PG collected the data and conducted interviews. MD and WC developed the questionnaire and conducted the analysis. QG, RG, and LJ gave advice on the statistical analysis and data processing and offered comments to modify the manuscript. JL contributed to the study design and supervised the process. All authors contributed to the article and approved the submitted version.

## Funding

This research was supported by the National Natural Science Foundation of China (Grant Nos. 72004165, 71774030, and 72104186), the Natural Science Foundation of Shandong Province (Grant No. ZR2020QG057), and the Postdoctoral Research Foundation of China (Grant No. 2020M681191).

## Conflict of Interest

The authors declare that the research was conducted in the absence of any commercial or financial relationships that could be construed as a potential conflict of interest.

## Publisher's Note

All claims expressed in this article are solely those of the authors and do not necessarily represent those of their affiliated organizations, or those of the publisher, the editors and the reviewers. Any product that may be evaluated in this article, or claim that may be made by its manufacturer, is not guaranteed or endorsed by the publisher.
